# In vitro cytotoxicity of *Aspilia pluriseta* Schweinf. extract fractions

**DOI:** 10.1186/s13104-021-05472-4

**Published:** 2021-02-09

**Authors:** Sospeter N. Njeru, Jackson M. Muema

**Affiliations:** 1 Department of Biochemistry, School of Health Sciences, Kisii University, P.O. Box 408-40200, Kisii, Kenya; 2 Department of Biochemistry, Jomo Kenyatta University of Agriculture and Technology (JKUAT), P.O. Box 62000-00200, Nairobi, Kenya; 3 Present Address: Division of Immunology, Paul-Ehrlich-Institute, Federal Institute for Vaccines and Biomedicines, Paul-Ehrlich-Straße 51-59, 63225 Langen, Hessen Germany

**Keywords:** Vero cells, Medicinal/herbal plants, Traditional/folk medicine, MTT assay, Selectivity index, Phytochemicals

## Abstract

**Objectives:**

We and others have shown that *Aspilia pluriseta* is associated with various biological activities. However, there is a lack of information on its cytotoxicity. This has created an information gap about the safety of *A. pluriseta* extracts. As an extension to our recent publication on the antimicrobial activity and the phytochemical characterization of *A. pluriseta* root extracts, here we report on cytotoxicity of tested solvent fractions. We evaluated the potential cytotoxicity of these root extract fractions on Vero cell lines by 3-(4,5-dimethylthiazol-2-yl)-2,5-diphenyltetrazolium bromide (MTT) assay.

**Results:**

We show that all solvent extract fractions (except methanolic solvent fractions) had cytotoxic concentration values that killed 50% of the Vero cells (CC_50_) greater than 20 µg/mL and selectivity index (SI) greater than 1.0. Taken together, we demonstrate that, *A. pluriseta* extract fractions’ earlier reported bioactivities are within the acceptable cytotoxicity and selective index limits. This finding scientifically validates the potential use of *A. pluriseta* in the discovery of safe therapeutics agents.

## Introduction

Plant-derived products and compounds have been used to treat and manage a wide range of diseases and infections since ancient times. The utilization of plant-derived products and compounds is favoured because these products and compounds exhibit fewer side effects, have improved efficacy and have reduced chances of developing resistance [1–5]. The bioactivity of plant extracts are a result of secondary metabolites, also called phytochemicals [3]. These phytochemicals are produced for normal plant defences. However, they inadvertently work against microbial systems and thus are often tapped for therapeutic interventions.

Here we extend the findings of our previous publications [5, 6] by looking at the safety of *Aspilia pluriseta* Schweinf. (*Asteraceae*) extract fractions in an in vitro system. *A. pluriseta* is a common herb in Kenya [5, 7], as well as in East, Central, and Southern Africa, [8, 9]. *A. pluriseta* is traditionally used to manage and treat wounds, cough, stomach illness, burn wounds, pimples, ears-, eye-, nose infections, kwashiorkor, fever, worms disorders, and diabetes mellitus with little or no scientific validation [7, 9–14]. Recently we have reported *A. pluriseta* selective antitubercular activity [5]. Other studies have reported *A. pluriseta* antiviral [9], antihelmintic [15], antimalarial, hypoglycaemic [7, 14, 16], molluscicidal [17] and complement modulating activities [18]. However, the scientific evidence of its pharmacological activity is not fortified by data on its cytotoxicity. We therefore aimed to fill this scientific information-gap using an in vitro cytotoxicity system. We report that, the *A. pluriseta* extract fractions (except methanol solvent fraction) have CC_50_ > 20 µg/mL, and SI > 1.0, which indicates that, *A. pluriseta* extract fractions are safe for use in drug discovery and that the reported bioactivity is not a result general toxicity.

## Main text

### Methodology

#### Plant material collection

Ethnopharmacological approach was used to identify the plant under study (*A. pluriseta*). This involved collecting information on *A. pluriseta* herbal use in the management and treatment of “strong coughs” and complicated respiratory infections from Mbeere community herbal practitioners. The gleaned information was further confirmed from documentation by Riley and Brokensha (1988) in *The Mbeere in Kenya *(ii)*, Botanical identity and use* [19]. *A. pluriseta* root samples were collected in an open community field, and the plant is not among the endangered plant species. Therefore, no prior permission was sought before the plant samples were collected. We collected the plant samples within GPS co-ordinates 0°46′27.0"S 37°40′54.9"E; −0.774156, 37.681908. Further authentication of plant sample identity was undertaken by Prof. S. T. Kariuki, a botanist at Egerton University, Kenya. A voucher specimen (number NSN2) was assigned and deposited at the same institution’s herbalium.

### Processing of plant samples

The plant materials were processed, extracted and finally fractionated as described in Njeru and Muema [5]. Briefly, root materials were cut into small pieces and allowed to air-dry in the dark at 23 ± 2 ℃ until they attained a constant weight. They were thereafter ground into fine powder with an electric miller (Retsch SR 200, Haan, Germany). Fifty grams of ground material was macerated in 200 mL methanol (Sigma Aldrich, St. Louis, USA) for 48 h. The extract was filtered out using Whatmann 1 filter paper, and the process repeated once more. Both filtrates were pooled together, and excess methanol evaporated from the filtrate by a rotor evaporator (Laborota 4000 efficient, Heidolph, Germany). The resulting dry extract was stored at −20˚C until use. To fractionate *A. pluriseta* root samples, we used organic solvents of increasing polarity (petro ether, dichloromethane, ethyl acetate and methanol respectively). Root powder (50 g) was macerated in 200 mL of petro ether solvent with intermittent shaking for 48 h. Thereafter, the extract was filtered out. Another 200 mL petro ether (PE) was added into plant material, and the process repeated after which the two filtrates were pooled together. The resulting marc was air-dried, after which it was further fractionated with solvents of increasing polarity (namely dichloromethane (DCM), ethyl acetate (EA), and finally methanol (MeOH) solvent in that order. The organic solvent fractions were concentrated with rotor evaporator as described before [5]. For assays, the organic solvent fractions were reconstituted into appropriate stock solutions with 100% dimethyl sulfoxide (DMSO), but diluted appropriately with culture medium so that the final DMSO concentration in the test sample was one percent, and therefore 1% DMSO was used as the negative control. The antitubercular activity, general antimicrobial activity, as well as the analytical characterization of phytochemicals of crude extract and solvent extract fractions evaluated here have been reported in our previous publications [5, 6].

### In vitro cytotoxicity test

An MTT assay previously described by Njeru, Obonyo [20] was followed to evaluate the toxicity of the *A. pluriseta* extract fractions on Vero cells (from African green monkey kidney cells (*Cercopithecus aethiops* epithelial cell line; ATCC CCL-81)). MTT assay is a colourimetric assay pegged on the ability of mitochondrial enzyme (succinate dehydrogenase) to reduce tetrazolium salt MTT to water-insoluble coloured substance (formazan) that is spectrophotometrically measurable [21, 22]. The amount of the formazan formed is directly proportional to the measure of cell viability. This is because only metabolically active cells can reduce MTT into formazan. The Vero cell line grown to 70–80% confluency in a medium (containing 100 mL DMEM, 10 mL fetal bovine serum (FBS), 1 mL penicillin–streptomycin, 1 mL amphotericin B, 1 mL L-glutamine and 0.1 mL gentamycin) was incubated in the presence of sample extract fractions at standard conditions (37 ℃ in 5% CO_2_) at 1.0 × 10^5^ cells/mL in a 96-well microtiter plate. The cells were exposed to decreasing concentrations of respective solvent extract fractions (250–0.24 μg/mL for petroleum ether and dichloromethane fractions; 500–0.49 μg/mL for ethyl acetate and methanolic fraction). Each sample concentration was tested in duplicates for 48 h. A post-exposure incubation of 4 h in 10 µL of 5 mg/mL MTT solution followed the addition of 100 µL acidified isopropanol (0.04 N HCl in isopropanol). The well plates were gently shaken for 5 min to dissolve the formazan in acidified isopropanol, and then optical density measured using ELISA Scanning Multiwell Spectrophotometer (LabSystems–Multiskan EX) at 562 nm using 690 nm as the reference wavelengths. The last column of microtiter well plate containing medium without plant solvent extract fractions, but with 1% DMSO, was included as the negative control. The percentage cell viability (%) was calculated at each concentration using the formula provided below [1, 20, 23].

$$ {\text{Cell viablity }}\left( \% \right) = \frac{{{\text{OD of sample}}_{562} {\text{ - OD}}_{690} \, }}{{{\text{OD of control}}_{562} {\text{ - OD}}_{690} }}*100 $$

Cytotoxic concentration values which represented the treatment concentration that kills 50% of the Vero cells (CC_50_), was determined by regression analysis. A particular plant solvent extract fraction was considered cytotoxic if it had CC_50_ of less than 20 μg/mL and selectivity index (SI) of less than 1.0 [1, 24, 25].

## Results

The cytotoxicity test was performed against Vero cells (from monkey kidney fibroblast cells) to ascertain the safety of *A. pluriseta* solvent extract fractions. We chose the Vero cell line as an ideal in vitro model for the study because of its sensitivity to toxicity, ease to culture, and it was readily available in our test facility. Additionally, Vero cells are recommended as a model to detect basal cytotoxicity [1, 26, 27]. In this study, we set a threshold of the cytotoxic concentration (CC_50_) below 20 µg/mL to be toxic, and above 20 µg/mL to be non-toxic as previously reported [1, 24, 25]. Our initial test for the cytotoxicity of the methanolic crude (cMeOH) extract revealed that the CC_50_ was within the acceptable toxicity limit (CC_50_ of 24.51) [6]. Therefore, we hypothesized that fractionation could help us identify active fractions that would not only maintain a strong bioactivity [5] but also be within the acceptable toxicity limits (CC_50_ > 20 µg/ml), and selectivity limits (SI > 1.0). Solvent extract fractionation gave us one fraction (MeOH at CC_50_ 14.36 µg/ml), which was cytotoxic. The PE fraction at CC_50_ 78.6 µg/ml, DCM fraction, at CC_50_ of 191.7 µg/ml, and EA fraction at CC_50_ > 500 µg/ml were all within the acceptable toxicity limit according to the set criteria (Table 1).

**Table 1:** Cytotoxicity of *A. pluriseta* solvent crude and fraction extracts

	_C_MeOH	PE	DCM	EA	MeOH
CC_50_ (µg/mL)	24.51	78.6	191.7	> 500	14.36

_C_MeOH, Crude methanolic extract; PE, Petroleum ether solvent fraction; DCM, Dichloromethane solvent fraction; EA, Ethyl acetate solvent fraction; MeOH, Methanol solvent fraction; CC_50_, Concentration that kills 50% of the cells

To determine the selectivity index (SI) of the solvent extract fractions, we divided their CC_50_ with their antitubercular MIC (all in µg/mL) (data published in [5]) as previously done by others [1, 24, 25]. The SI ranged from 1.1488 to 80 (Table 2), which according to Afagnigni, Nyegue [1] and Mongalo, McGaw [24], suggested that the *A. pluriseta* extract fractions were not toxic, or, in the case of MeOH solvent extract fraction, that it exhibited cytotoxicity and antitubercular activity almost equally [1, 24, 25].

**Table 2:** Selectivity index of *A. pluriseta* solvent extract fractions

	PE	DCM	EA	MeOH
Selectivity index	3.144	7.668	80	1.1488

PE, Petroleum ether solvent fraction; DCM, Dichloromethane solvent fraction; EA, Ethyl acetate solvent fraction; MeOH, Methanol solvent fraction

## Discussion

Although plants’ contribution to new and novel leads for therapeutic drug development has been accepted for a long time now, it is currently a known fact that plant extracts are not always safe [20, 28]. The cytotoxicity of many herbal-derived products is a potential source of more deleterious side effects to subjects. It is, therefore, imperative to determine whether plant extracts and products showing potential drug activities are active within the acceptable toxicity and selectivity index limits [1, 24, 25, 29, 30]. Interestingly, we found that the crude extract and solvent extract fractions (except methanolic solvent extract fraction) demonstrated activity within the acceptable cytotoxicity limit (Table 1). Furthermore, all the solvent fractions had selectivity index of > 1.0, which further confirms that the solvent extract fractions are not toxic and hence the reported bioactivity in [5] was not due to basal metabolic toxicity, or in the case of MeOH solvent extract fraction, the bioactivity and cytotoxicity are almost the same [24].

## Conclusion

Our findings demonstrate that *A. pluriseta* root solvent extract fractions’ previously reported bioactivity is within acceptable cytotoxicity and selectivity index limit, and thus provide a potential source for safe drug candidate(s).

## Limitation

It is important to note that the in vitro cytotoxicity results do not always equate to in vivo toxicity. This may be attributed to physiological, anatomical pharmacodynamic, and pharmacokinetic considerations in living animals and cell culture [1, 24, 29]. Therefore, there is a need for further in vivo toxicity assessment of the extract fractions. In this study, we evaluated the cytotoxicity of solvent extract fractions. However, it will be interesting in the future to isolate the active phytoconstituents (which we previously reported to be present in the tested extracts [5, 6]) and test their individual biological and cytotoxicity effects.

## Data Availability

All data generated or analyzed during this study are included in this published article.

## Abbreviations

PE: Petroleum ether
DCM: Dichloromethane
EA: Ethyl acetate
MeOH: Methanol
cMeOH: Crude methanolic extract
MTT: 3-(4,5-Dimethylthiazol-2-yl)-2,5-diphenyltetrazolium bromide
CC_50_: Cytotoxic concentration values that killed 50% of the Vero cells
SI: Selective index
